# Mature Cystic Teratoma Combined With Autoimmune Limbic Encephalitis Mimicking Functional Ovarian Cyst

**DOI:** 10.7759/cureus.26812

**Published:** 2022-07-13

**Authors:** Jong Ha Hwang, Bo Wook Kim

**Affiliations:** 1 Obstetrics and Gynecology, International St. Mary’s Hospital, Catholic Kwandong University College of Medicine, Incheon, KOR

**Keywords:** functional cyst, ovarian teratoma, autoimmune limbic encephalitis, nmdar receptor, mature cystic teratoma

## Abstract

Autoimmune limbic encephalitis (ALE) associated with an anti-N-methyl-D-aspartate receptor (NMDAR) is a rare but occasionally fatal condition that could be accompanied by ovarian teratoma. We report a case of a 27-year-old woman with ALE combined with a mature cystic teratoma that looks like a functional cyst in imaging studies. A single port access laparoscopic left oophorectomy was performed. On the 154th postoperative day, symptoms were fully recovered. Teratoma detection and tumor removal are critical for the management of patients diagnosed with or suspected of ALE.

## Introduction

Autoimmune limbic encephalitis (ALE) is a rare neurological disease that is mainly associated with ovarian teratoma, although it occurs mostly in conjunction with small-cell lung tumors and testicular tumors. Its pathogenesis is presumed to be an autoimmune mechanism. Progressive neurological symptoms, such as memory impairment, emotional disturbance, and cognitive impairment, are found prior to the detection of the primary tumor, which makes it difficult to differentiate from a primary psychiatric disease [1]. Removal of tumors is important for treatment. However, in some cases, tumors associated with ALE are not detected on radiological examination.

## Case presentation

A 27-year-old unmarried woman was admitted to the outpatient department of neurology due to rigidity and dyskinesia. She had no specific findings in her past history, and the normal healthy patient had only a few meals due to nausea and vomiting for the past week. Afterward, she was unable to discern the time and later experienced auditory hallucinations and memory impairment. Moreover, deterioration of her cognitive function was observed. Therefore, she was admitted to the emergency room of the hospital. She started taking antipsychotic medication from a psychiatrist, but two days later, she started showing signs of rigidity and dyskinesia.

**Figure 1:**
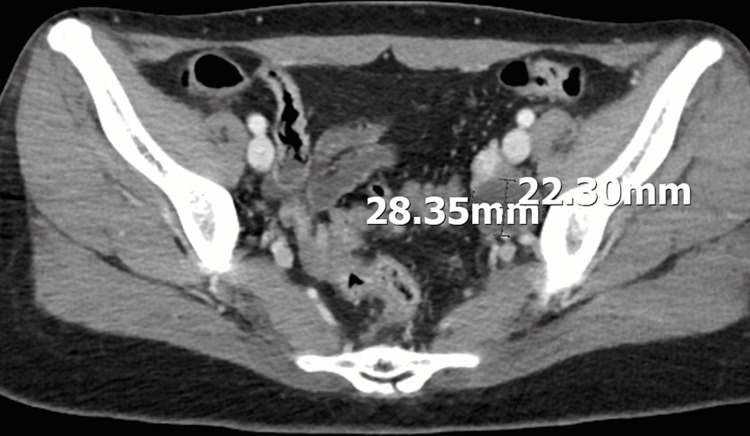
Pelvic CT showed a tumor that looked like a functional left ovarian cyst, which measured about 2.8 cm.

Brain computed tomography (CT), brain magnetic resonance imaging (MRI), and angiography did not reveal any abnormality. The electroencephalogram (EEG) was normal. Cerebrospinal fluid (CSF) tapping showed mild pleocytosis (white blood cell 30/mm^3^ and lymphocyte 80%). Ceftriaxone and acyclovir were administered under suspicion of limbic encephalitis caused by inflammation such as herpes simplex virus (HSV). However, a gradual deterioration in her health was observed along with behavioral changes, hypoventilation, general rigidity, and repetitive generalized tonic-clonic seizures. She was transferred to the intensive care unit and intravenous immunoglobulin and methylprednisolone were administered under suspicion of limbic encephalitis. Since she also had convulsions, anticonvulsants such as phenobarbital, carbamazepine, sodium valproate, and fosphenytoin were administered to her. Even after treatment with drugs, the oxygen saturation level decreased, so the patient was put on a ventilator on the fourth day of hospitalization. Under the suspicion of ALE, an abdominal pelvic CT was performed. A tumor that looked like a simple cyst arising from the left adnexa, measuring about 2.8 cm, was found on CT (Figure 1).

**Figure 2:**
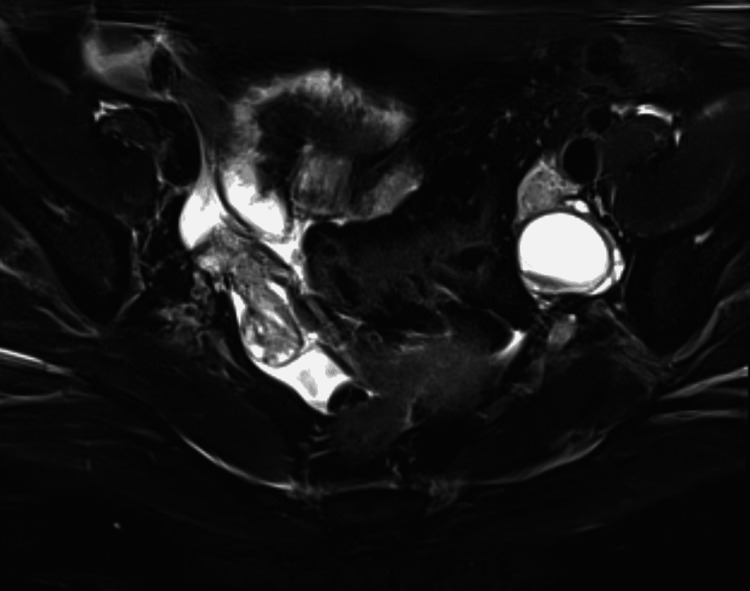
Pelvic MRI showed an unilocular cystic lesion with focal calcification and a small amount of sludge inside the left ovarian cyst. However, restricted diffusion, abnormal enhancement, and heterogeneous composition were not observed.

She was referred to a gynecologist, and pelvic ultrasonography and MRI were performed on her. An unilocular cystic lesion with focal calcification was seen inside the left ovary cyst, and a small amount of sludge was present in the dependent portion in pelvic MRI (Figure 2).

Although mature cystic teratoma could not be excluded, the possibility of a functional cyst was high. CA 125 and HE 4 were 6.2 U/mL and 75.1 pmol/L, respectively, which corresponded to the high risk of epithelial ovarian cancer in premenopausal women. The squamous cell carcinoma antigen (TA-4) was increased to 2.1 ng/mL, which was higher compared to the reference value (0-1.5 ng/mL).

HSV type I/II IgM and HSV type I/II real-time PCR were negative in CSF and serum. PCR results for Tb/NTM, cytomegalovirus, Epstein-Barr virus, and varicella zoster were all negative. Since the patient's condition did not improve, ALE was suspected, and after discussing with the family about the potential for malignant or functional ovarian cyst and the benefits of surgery, a single port access laparoscopic left oophorectomy (LSO) was performed. There was a little cloudy yellow fluid pooled inside the cyst, but no solid matter or hairs were observed. Histopathological examination revealed a mature cystic teratoma and a para tubal cyst. After surgery, due to persistent orofacial dyskinesia such as jaw opening and closing, chewing, and lip pouting, she was maintained on a ventilator. The antibody test result against the N-methyl-D-aspartate receptor (NMDAR) antibody in CSF was positive, which was reported on the third postoperative day. The antibodies against AMPA, DPPX, LSI1, CASPR2, and GABA-B were not detected. Postoperatively, rituximab was administered.

After 18 days of LSO, a tracheostomy was performed due to repeated seizures, but the patient's condition gradually improved thereafter. On the 69th day after surgery, she was transferred from the intensive care unit to the general ward. She showed excellent improvement and was now able to move independently without further seizures. She was eventually discharged 98 days after surgery and had a tremor in her left hand at the time of discharge. At the outpatient visit on the 154th postoperative day, her symptoms were fully recovered.

## Discussion

ALE was first described in 2007 [2]. If the CSF has pleocytosis and has neurological symptoms such as hallucinations, memory impairment, emotional disturbance, cognitive impairment, and convulsions, encephalitis is suspected and inflammation is treated. If there is no response to the anti-inflammatory treatment, autoimmune antibody testing such as anti-NMDAR should be performed in consideration of ALE. ALE can occur in small-cell lung cancer and immature teratoma of the testis, but most patients are female and are associated with ovarian teratoma. Teratomas are known as benign ovarian tumors and less than 1% of teratomas are immature teratomas. When teratoma is associated with ALE, the probability of immature teratoma is relatively high [3-5]. Dalmau et al. [3] analyzed 100 cases of anti-NMDAR receptor encephalitis and found that 91% of them were female. Among them, ovarian and immature teratomas were found in 49 and 14 cases (28.6%), respectively. Li et al. [4] reviewed eight cases of anti-NMDAR encephalitis associated with bilateral teratoma, and among them, three cases had immature teratoma. ALEs associated with teratomas are most common in women of childbearing age in their 20s and early 30s but have also been reported in five-year-old girls and up to 76-year-old women.

Most cases with ALE show lymphocytic pleocytosis with CSF tapping. Slow activity is often seen in EEG. Although the clinical features of ALE are similar to those of herpes simplex encephalitis, it has a relatively low fever and severe psychotic symptoms and can show bilateral temporal lobe involvement on brain MRI [6]. According to case reports of teratoma associated with ALE, 50% of these teratomas were larger than 6 cm in size, but teratomas with a size of 1 cm were also observed [7]. This case was a simple ovarian cyst that looked like a functional cyst, with a diameter of less than 3 cm. Gynecological ultrasonography and pelvic MRI showed only a few suspicious lesions.

Abdul-Rahman et al. [8] conducted a pelvic MRI on a 25-year-old woman who was positive for anti-NMDAR antibodies in CSF and serum and was suspected to have a teratoma. The radiological examination revealed a 1.9 cm hemorrhagic cyst on the right side and a 2.6 cm simple ovarian cyst on the left side, but no teratoma was found. Positron emission tomography (PET) scans or tumor markers such as CA 125 and CEA were also negative. Although the authors were not sure of the teratoma, bilateral salpingo-oophorectomy was performed on the 29th day of admission after consulting with the family about the risk of early menopause and the possibility of neurological recovery. They harvested ovarian tissue for cryopreservation before surgery. Pathological examination revealed a mature cystic teratoma with mature neuroglial elements in the left ovary. Johnson et al. [9] performed a CT scan when a 35-year-old woman with nonconvulsive status epilepticus and anti-NMDAR receptor was unresponsive to immunotherapy. A hemorrhagic cyst was found on CT and oophorectomy was performed five months after initiation of treatment. Pathological examination revealed ovarian teratoma, after which the patient recovered. Boeck et al. [10] performed CT and PET under similar circumstances, and there were no specific findings. Ultrasonography showed a minimal suspect lesion in the right ovary, so a laparoscopic bilateral ovarian biopsy was performed, but there were no specific findings. When various immunotherapies were ineffective, a right oophorectomy was performed at 11 months of treatment. Pathological examination confirmed a teratoma with partial neuronal differentiation. The patient progressed to a point where she could communicate in short sentences. Table 1 summarizes examples in which ALE was suspected but the teratoma was not clearly visible on radiologic examination.

**Table 1:** A literature review of cases of mature cystic teratoma associated with autoimmune limbic encephalitis that was not clearly visible on radiologic examination. Abbreviation: EEG, Electroencephalography; MRI, Magnetic resonance image; CT, Computed tomography; Sono, Ultrasonography; PET, Postitron emission tomography. ^a^Harvesting of ovarian tissue for cryopreservation was done before surgery

Published Year & Author	Age	Symptom	Preoperative radiologic examination	Radiologic finding	Operative name	Operative timing	Outcome	Recovery time after surgery	EEG	Preoperative anti-NMDAR antibody confirmation	Preoperative immuotherapy
This case, Hwang et al.	27	Auditory hallucination	MRI, CT, SONO	Left functional cyst (2.8 cm)	Laparoscopic left salpingo-oophorectomy	7 days after admission	Completely recovered	5 months	Normal	No	immunogloblulin, methyprednisolone
2010, Johnson et al. [9]	35	Persistent nonconvulsive status epilepticus	CT	Hemorrhagic cyst	oophorectomy	5 months in pentobarbital-induced burst suppression	Partially recovered	6 months	Cyclical pattern of moderate to high voltage	Yes	immunogloblulin, rituximab, cyclophosphamide
2013, Boeck et al. [10]	34	Hyperkinesias, autoimmune dysfunction, hypoventiation, epileptic status	CT, PET, SONO	Right minimal suspect lesion	Right oophrectomy	11 months after initial presentation	Partially recovered	12 months	Unknown	Yes	Immnuoglobulin, plasma exchange, rituximab, cyclophosphamide,
2016, Abdul-Rahman et al. [8]	25	Generalized tonic-clonic seizure	MRI, PET	Right hemorrphagic cyst (1.9 cm), Left ovary simple cyst (2.6 cm)	Laparoscopic bilateral salpingo-oophorectomy^a^	29 days after admission	Completely recovered	3 months	Periodic lateralized epileptiform discharges, slow wave	Yes	immunogloblulin, methyprednisolone, plasmapheresis

## Conclusions

In the present case study, we performed laparoscopic right salpingo-oophorectomy under suspicion of ALE before NMDAR antibody results were available because the symptoms worsened despite the treatment with an antiviral agent and immunotherapy. When anti-NMDAR antibodies are related to ovarian teratoma, surgical removal is recommended as early as possible because early removal of the tumor is associated with a better neurological outcome and a shorter recovery time. Although ovarian cystectomy or oophorectomy is possible, unilateral salpingo-oophorectomy was performed due to microscopic tissue of the teratoma that is likely to remain after ovarian cystectomy and the risk of immature teratoma. The neurological symptoms of ALE can be fatal, and removal of the teratoma can help relieve symptoms. Therefore, if the patient does not respond to an antiviral agent and immunotherapy, a prompt multidisciplinary approach is required and oophorectomy or salpingo-oophorectomy should be considered as early as possible, even if the teratoma is not evident on radiological examination.
